# Is Height^2.7^ Appropriate for Indexation of Left Ventricular Mass in Healthy Adolescents? The Importance of Sex Differences

**DOI:** 10.1161/HYPERTENSIONAHA.121.17109

**Published:** 2023-08-07

**Authors:** Hannah C. M. Taylor, Nishi Chaturvedi, George Davey Smith, Diana L. S. Ferreira, Abigail Fraser, Laura D. Howe, Alun D. Hughes, Debbie A. Lawlor, Nic J. Timpson, Chloe M. Park

**Affiliations:** MRC Unit for Lifelong Health and Ageing, University College London, United Kingdom (H.C.M.T., N.C., A.D.H., C.M.P.).; Oxford Population Health (NDPH), University of Oxford, United Kingdom (H.C.M.T.).; Department of Epidemiology and Biostatistics, School of Public Health, Imperial College London, United Kingdom (H.C.M.T.).; MRC Integrative Epidemiology Unit, University of Bristol, United Kingdom (G.D.S., D.L.S.F., A.F., L.D.H., D.A.L., N.J.T.).; Bristol Population Health Science Institute, Bristol Medical School, University of Bristol, United Kingdom (G.D.S., D.L.S.F., A.F., L.D.H., D.A.L., N.J.T.).

**Keywords:** adiposity, adolescents, body composition, hypertrophy, left ventricular

## Abstract

**BACKGROUND:**

Left ventricular mass (LVM) is an important predictor of cardiovascular risk. In adolescence, LVM is commonly indexed to height^2.7^, although some evidence suggests that this may not fully account for sex differences.

**METHODS:**

We investigated appropriate allometric scaling of LVM to height, total lean mass, and body surface area, in a UK birth cohort of 2039 healthy adolescents (17±1 years). Allometric relationships were determined by linear regression stratified by sex, following log transformation of x and y variables [log(y)=a+b×log(x)], b is the allometric exponent.

**RESULTS:**

Log (LVM) showed linear relationships with log(height) and log(lean mass). Biased estimates of slope resulted when the sexes were pooled. The exponents were lower than the conventional estimate of 2.7 for males (mean [95% CI]=1.66 [1.30–2.03]) and females (1.58 [1.27–1.90]). When LVM was indexed to lean mass, the exponent was 1.16 (1.05–1.26) for males and 1.07 (0.97–1.16) for females. When LVM was indexed to estimated body surface area, the exponent was 1.53 (1.40–1.66) for males and 1.34 (1.24–1.45) for females.

**CONCLUSIONS:**

Allometric exponents derived from pooled data, including men and women without adjustment for sex were biased, possibly due to sex differences in body composition. We suggest that when assessing LVM, clinicians should consider body size, body composition, sex, and age. Our observations may also have implications for the identification of young individuals with cardiac hypertrophy.

**NOVELTY AND RELEVANCE**

**What Is New?**

This study investigates sex-stratified allometric scaling of left ventricular mass (LVM) to height, lean mass, and body surface area in addition to the detection of left ventricular hypertrophy (LVH) using different clinical cutoffs, in over 2000 seventeen-year olds.

**What Is Relevant?**

Indexing by any measure of body size may be biased if data from both the sexes are pooled.

In older adolescents, (1) it may be inappropriate to normalize LVM to body surface area; (2) indexation of LVM to lean mass (when available) may be a better option than height; and (3) if height is used, our data suggest height^1.7^ may be a more appropriate index than height^2.7^.

We highlight important inconsistencies between the detection rate of LVH when different clinical cutoffs are applied. Such inconsistencies have the potential to result in high-risk individuals being misclassified.

**Clinical/Pathophysiological Implications**

In contrast with common clinical practice, a gender-blind method when normalizing LVM to body size may be inappropriate, and we advocate that body size, composition, sex, and age should be taken into account when indexing LVM.

Further research is required for defining the most appropriate clinical cutoff for detection of LVH in late adolescence.

**See related article, pp 2043-2045**

Cardiovascular disease is the leading cause of mortality in Europe, accounting for 45% of all deaths.^1^ Elevated left ventricular mass (LVM) is an important prognostic biomarker and predicts cardiovascular morbidity and mortality, independent of traditional cardiovascular risk factors.^2^ The measurement of LVM can also diagnose inherited cardiac diseases, such as familial hypertrophic cardiomyopathy.^3^ However, it is important that the relationship between LVM and body size is accounted for appropriately when determining whether an individual has left ventricular hypertrophy (LVH).^4^

In adults, LVM is commonly indexed to body surface area (BSA),^4^ although this method has been criticized as it underestimates LVH, especially in those who are overweight, obese, or who have obesity-related diseases such as hypertension.^5^ Due to these shortcomings, indexing LVM to height raised to allometric powers such as 1.7^6^ and 2.7^5^ has been advocated in adults, because it approximates lean body mass.^7^ Height-based indexation methods are generally considered to be better at accounting for body size and detecting LVH than BSA and are more inclusive of different ethnicities, particularly when predicting events in obese patients.^6^ The use of height-based exponents has also been described as being the most promising clinical method for scaling myocardial mass to body size.^8^ In young people, height^2.7^ is most widely used, but in adults this approach has been reported not to account sufficiently for sex differences, and may lead to the misclassification of subjects with respect to the presence of LVH.^6^ However, it is unclear whether findings in middle-aged or elderly cohorts can be generalized to young people^9^ and consequently the best method for indexing LVM to body size remains uncertain in younger cohorts.

It has been proposed that lean mass may be superior to height or BSA for indexing LVM in both adults and children,^10–14^ as it is not disproportionately affected by fat mass.^15^ However, it is more difficult to measure lean mass accurately using routine practice data and the predictive value of LVM indexed to lean mass compared with other indexes is limited. Lastly, the importance of accounting for sex differences when considering body size has been highlighted previously.^16^

Due to the lack of clarity in the literature as to the best anthropometric measurement to which to index LVM in late adolescence, we investigated the impact of indexing LVM to height, lean mass, and BSA in a large community-based cohort of UK adolescents. We considered the roles of (a) sex, (b) body composition, and (c) the impact of different indexing methods on LVH classification.

## METHODS

### Data Availability

The authors declare that all supporting data are available within the article (and its Supplemental Material).

### Sample and Study Design

**Figure 1.**
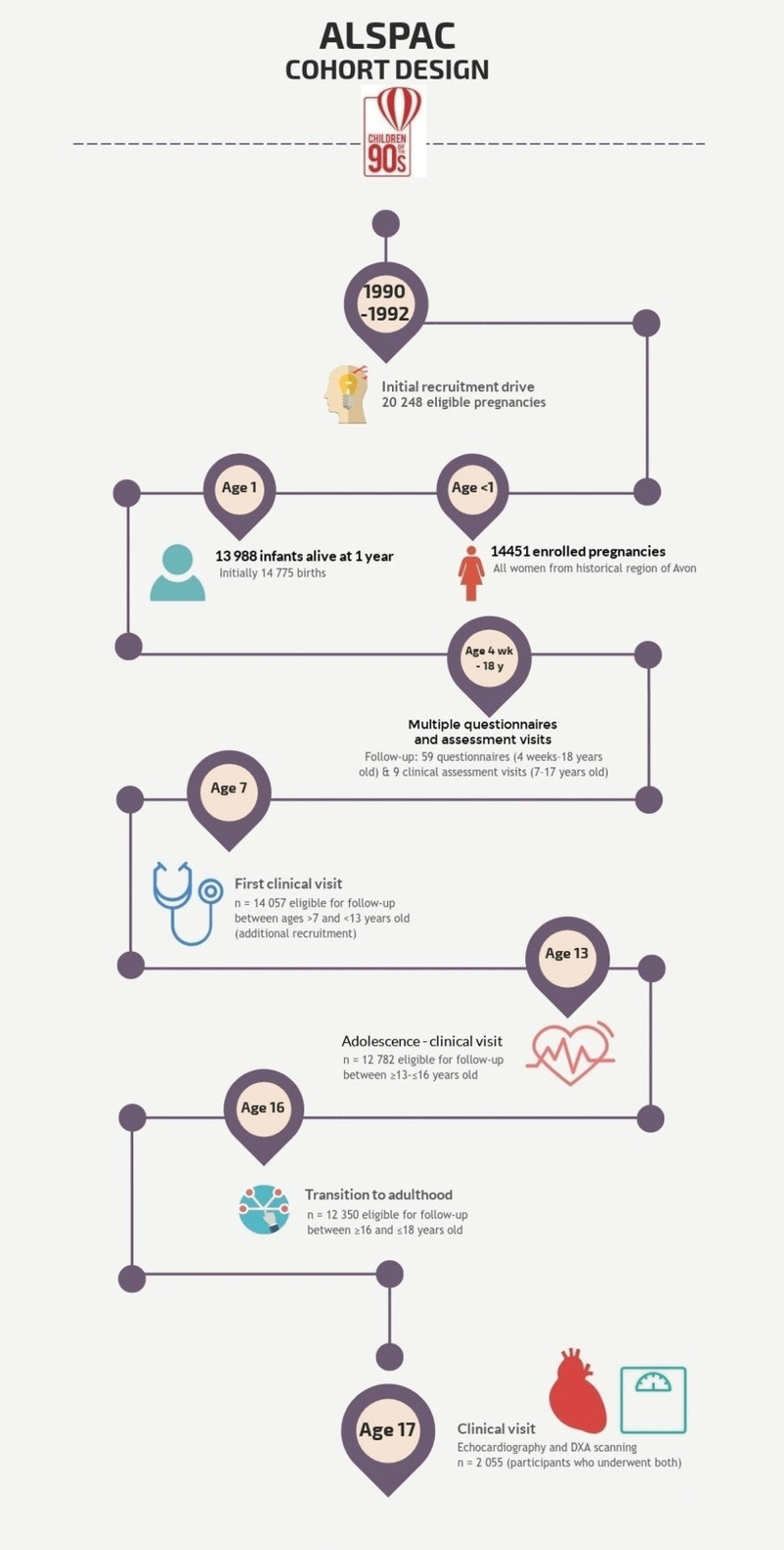
Original infographic highlighting key clinical visits and features of the ALSPAC (Avon Longitudinal Study of Parents and Children) cohort design.

Participants were drawn from ALSPAC (Avon Longitudinal Study of Parents and Children), a prospective population-based birth cohort study. The study was established in 1991 and recruited 14 541 pregnant women with expected dates of delivery between April 1, 1991 and December 31, 1992 resident in the South West of the UK (http://www.bris.ac.uk/alspac/researchers/data-access/data-dictionary). Of their offspring, 13 988 infants have been followed up, at intervals, participating in over 90 questionnaires and 10 clinical assessment visits. A large variety of data are available, covering a range of behavioral and biological factors. Basic details of the cohort are shown in Figure 1 and further details have been described elsewhere.^17^

### Focus Clinic at 17

At age 17 years, all study participants were invited to attend a clinical examination that included anthropometrics, echocardiography, and dual energy x-ray absorptiometry. A total of 5215 participants attended the clinic, all of whom provided written informed consent, and ethical approval for the study was obtained from the ALSPAC Law and Ethics Committee and the Local Research Ethics Committees.

### Echocardiography

Due to the design of the focus@17 clinic, only ≈1 in 2 of the total attendees (pseudorandom sample) underwent echocardiography. Two-dimensional (2D) echocardiography was performed using a Phillips HDI 5000 ultrasound machine equipped with a P4-2 Phased Array ultrasound transducer by one of the 2 echocardiographers, in accordance with the American Society of Echocardiography guidelines.^4^ Measures of LV wall thickness and chamber dimension were measured in the parasternal long axis view and used to calculate LVM using Devereux’s formula.^18^ Individuals with a history of heart disease, hypercholesterolaemia, diabetes, or who were pregnant were excluded from analyses. To determine the n (%) of participants with LVH, we applied the ASE cutoffs for unindexed and BSA-indexed results (both linear and 2D^4^). For height^2.7^, we used the cutoffs proposed by De Simone et al,^5^ and for height^1.7^, those used by Chirinos et al.^6^ Although the latter study used older participants aged 35 to 84 years, this analysis was well powered and our initial analyses suggested that the height^1.7^ exponent might be more appropriate for use in late adolescence than the height^2.7^ exponent.

### Anthropometry, Blood Pressure, and Blood Analysis

Height (rounded to the nearest centimeter) was measured using a Harpenden stadiometer (SECA 13, Birmingham, UK) and body mass (to the nearest 0.1 kg) was recorded using a Tanita scale (Marsden M-110, Rotherham, UK). Participants were weighed wearing lightweight clothing and without shoes. Body mass index (BMI) was calculated as kilogram per meter squared and BSA was calculated using the DuBois formula: BSA=0.007184×[height (cm)^0.725^]×[weight (kg)^0.425^]. Overweight and obese were defined as BMI >25 kg/m^2^ and >30 kg/m^2^, respectively. Body composition was assessed by dual energy x-ray absorptiometry using a Lunar Prodigy narrow fan-beam densitometer, from which total body fat mass and total lean mass, in kilograms, were quantified.

Seated systolic blood pressure and diastolic blood pressure were measured using an Omron 705 IT oscillometric blood pressure monitor in accordance with the European Society of Hypertension guidelines.^19^ The final 2 of 3 readings were averaged and used in analyses. Venous blood was drawn and analyzed for total cholesterol, high-density lipoprotein cholesterol, and triglycerides.

### Statistical Analyses

All statistical analyses were performed using Stata SE 15.1 and 17.0 (StataCorp LLC, College Station, TX). Participant physical and socioeconomic characteristics are reported as mean±SD, or median (interquartile range) for skewed data and n (%) for categorical data. Results of other statistical analyses are presented as mean (95% CIs). Comparisons between those with and without measured LVM were made using a 2 sample *t* test.

Multivariable linear regression was used to assess differences in LVM stratified by sex, after adjusting for either height/lean mass or BSA. Age was investigated as a confounder but, unsurprisingly given the narrow age range studied, did not affect the coefficients and has been omitted from the analyses for simplicity. Allometric relationships for height were determined using linear regression, following log transformation of x and y variables [ln(y)=a+b×ln(x; where b is the allometric exponent and a is the log of the normalization constant)]. Assumptions of linearity and homoscedasticity of residuals were checked for each model using residual plots, Breusch-Pagan/Cook-Weisberg, White and Szroeter tests. Where there was evidence of homoscedasticity, bias corrected 95% CIs were calculated using bootstrapping with 200 repetitions. Sex×b and fat mass×b interactions were investigated and due to the impact of sex and body size on LVM, analyses were stratified by sex. Further stratification by both gender and fat mass quartile (kilogram) was also undertaken. Detection of LVH using different cutoffs was quantified by deriving simple summary statistics and interrelater agreement was assessed using the kappaetc command.

## RESULTS

Participant characteristics are presented in Table 1. Forty-five percent were male and on average, males were taller and heavier with greater lean mass, less fat mass, increased LVM, and higher systolic blood pressure than females.

**Table 1.**
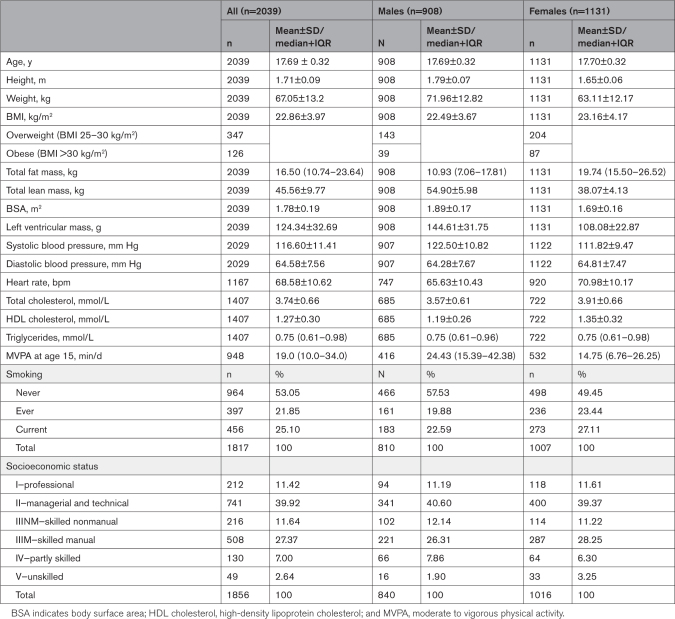
Baseline Characteristics of Participants With Measured Left Ventricular Mass

A comparison of those with and without echocardiography (Table S1) indicated that the individuals who did not undergo echocardiography had marginally higher heart rates, were more likely to smoke, and were from a slightly more unfavorable socioeconomic background. However, the groups were similar in terms of proportion of women, height, body size, and fat composition within sex strata.

### Effect Modification

Sex modified the associations between LVM and height (*P*=0.04), lean mass and BSA (both *P*<0.001; Table S4). When both sexes were pooled together, there was no evidence of modification of these associations by fat mass; however, when the sexes were considered separately, there was evidence that fat modified associations between LVM and lean mass (*P*<0.001) and BSA (*P*<0.001) in males only (allometric relationships between LVM and height, lean mass and BSA, stratified by both sex and quartiles of fat mass can be seen in Table S2).

### Allometric Relationships Between LVM and Height, Lean Mass and BSA

**Figure 2.**
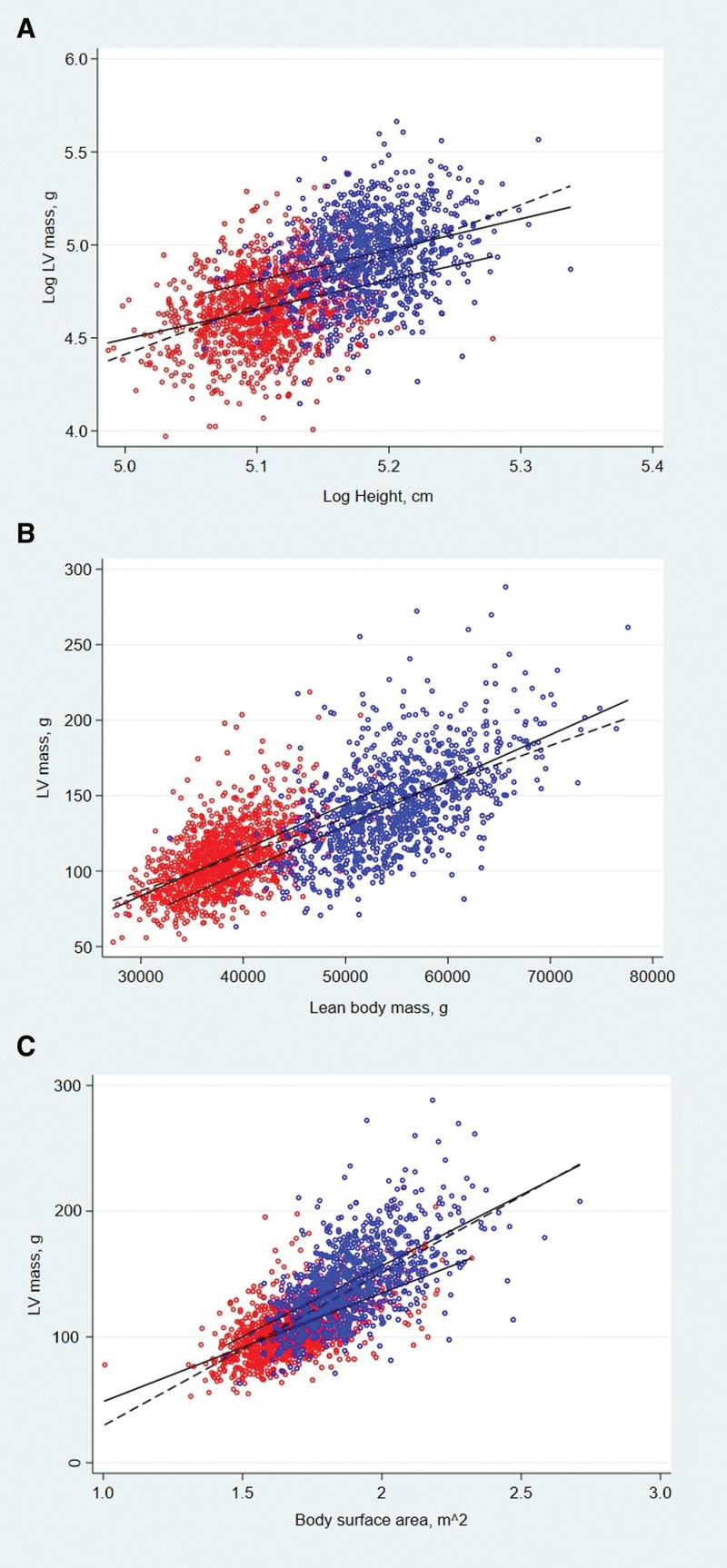
**Relationships between height, lean mass and body surface area and left ventricular (LV) mass, in males (blue circles) and females (red circles). A**, Relationship between height (log-transformed) and LV mass (log-transformed). **B**, Relationship between lean mass and LV mass (unlogged). **C**, Relationship between body surface area and LV mass (unlogged). Solid black lines indicate the gradient for each sex separately, while the dashed line indicates the gradient for the pooled group.

Figure 2A through 2C show the relationships between log(LVM) and log(height; Figure 2A), untransformed lean mass (Figure 2B) and untransformed BSA (Figure 2C). The association between LVM and height was allometric, the association with lean mass was linear, and the association with BSA was also allometric. After accounting for height (unlogged), men had on average 20.02 (16.67–23.37) g higher LVM than females. Height explained comparatively little of the variance in LVM. For the pooled group, r^2^=0.32, but in males and females separately this value decreased considerably (r^2^=0.08 for each sex). When both sexes were analyzed together, the allometric exponent relating LVM to height was 2.68 (2.51–2.85). When regression was performed in males and females separately, the height exponents were 1.66 (1.30–2.03) in males and 1.59 (1.27–1.90) in females (Figure 2A; Table 2). In addition, we derived mean values (with tolerance intervals [confidence level 0.95; coverage probability 0.90]) for LVM indexed to height^1.7^. These were 53.81 (25.01–103.93) and 46.11 (27.45–64.77) for males and females, respectively, with the 95% CIs incorporating the previous cutoffs suggested by Chirinos of 81 g/m^1.7^ for males and 60 g/m^1.7^ for females.^6^

**Table 2.**
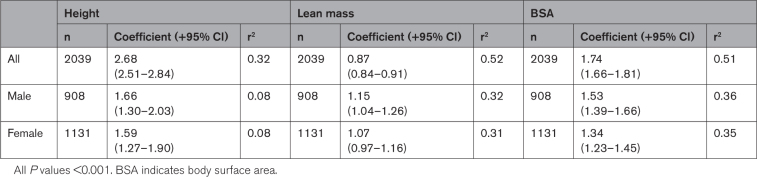
Allometric Relationships Between Left Ventricular Mass and Height, Lean Mass and BSA, Stratified by Sex and Presented With 95% CIs and r^2^ Values

The allometric exponents obtained from the relationship of log(LVM) to log(lean mass) was 0.87 (0.84–0.91) for all participants. Sex-stratified allometric analysis of the relationship between log(LVM) and log(lean mass) gave allometric exponents of 1.15 (1.04–1.27) for males and 1.07 (0.97–1.16) for females (Table 2). Plots of the sex-stratified log transformed data are shown in Figures S2 and S3). Because these estimates were close to unity, linear regression models were used in subsequent analyses of the relationship between LVM and lean mass (Figure 2). After adjustment for lean mass, women had on average 14.07 (10.25–17.90) g higher LVM than males. Lean mass explained much more of the variance in LVM than height, both when all participants were pooled (r^2^=0.52) and when males (r^2^=0.32) and females (r^2^=0.31) were considered separately.

In the model that adjusted for BSA, males had on average 16.31 (14.03–18.60) g higher LVM than females (Figure 2C) and the exponents were 1.74 (1.67–1.81), 1.53 (1.40–1.66), and 1.34 (1.24–1.45) for the group, males and females, respectively (Table 2). Interestingly, BSA accounted for approximately the same amount of variance in LVM as lean mass in the pooled group (r^2^=0.51), and for slightly more of the variance in males (r^2^=0.36) and females (r^2^=0.35) than lean mass.

Additional sensitivity analyses were performed to check whether the sex-specific exponents for height, lean mass, and BSA were much altered after the removal of (1) participants with obesity (BMI 30 kg/m^2^) and (2) participants who were overweight or obese (BMI >25 kg/m^2^; Tables S5 and S6). When compared with the estimates derived when all 2039 participants were retained, the removal of the more adipose participants did not have major effects on the estimates of the allometric exponent. The 95% CIs also remained similar.

### The Impact of Different Indexing Methods on Categorization of LVH

**Figure 3.**
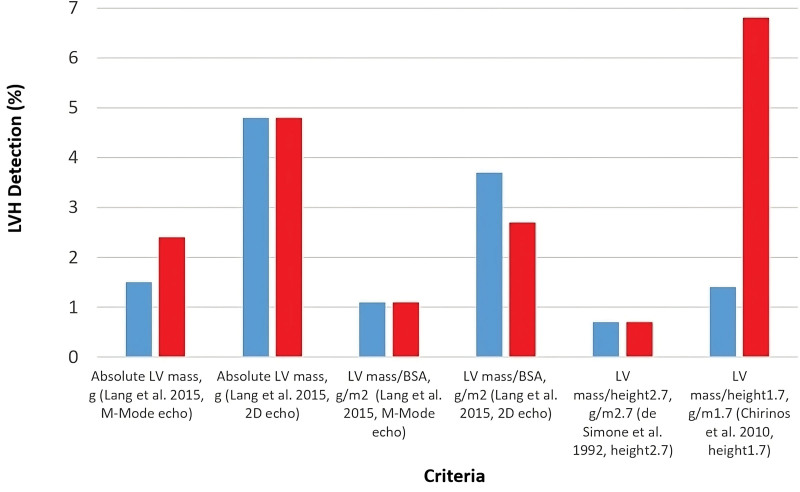
**Percentages of males (blue) and females (red) identified as having left ventricular hypertrophy (LVH), upon application of different recommended cutoffs.** LVH was detected using the following criteria: for absolute LV mass (M-mode/linear, Lang et al. 2015^3^), >224 g (males), >162 g (females); for absolute LV mass (2D echo, Lang et al. 2015^3^), >200 g (males), >150g (females); for LV mass/body surface area ([BSA]; M-mode/linear, Lang et al. 2015^3^), >115 g/m^2^ (males), >95 g/m^2^ (females); for LV mass/BSA (2D echo, Lang et al. 2015^3^), >102 g/m^2^ (males), >88 g/m^2^ (females); for LV mass/height^2.7^ (de Simone et al. 1992^5^), >50 g/m^2.7^ (males), >47 g/m^2.7^ (females); for LV mass/height^1.7^ (Chirinos et al. 2010^6^), >81 g/m^1.7^(males), >60 g/m^1.7^(females).

Figure 3 compares different clinical cutoffs in terms of prevalence of LVH, taken from.^4–6^ The overall range of detection was variable, with 0.7% to 4.8% (6–44 participants) of males and 0.7% to 6.8% (8–77 participants) of females identified as having LVH. The Fleiss’ kappa results for males (κ=0.38 [95% CI, 0.26–0.49]) and females (κ=0.40 [95% CI, 0.32–0.47]) indicated that there was only moderate agreement between the schemes. Although LVH detection was similar between each sex, LVM indexed to BSA and LVM indexed to height^2.7^, there was a very large discrepancy between the level of detection in males (1.4%) and females (6.8%) for LVM indexed to height^1.7^.

## DISCUSSION

We report findings that question the commonly used approach of normalizing LVM to height^2.7^ to account for body size in childhood and adolescence. In addition, using data from over 2000 seventeen-year-olds from ALSPAC, we show that pooling measurements from both sexes results in biased estimates of the relationship between LVM and measures of body size. Our findings indicate that sex may be an important factor to refine our understanding of LVH-related cardiovascular risk.

Our sex-stratified analyses show that the allometric relationship between LVM and height has an allometric exponent of ≈1.5 to 1.7 for both sexes. The slightly larger exponent we found for males (1.66) compared with females (1.59) is in keeping with other findings that boys have larger indexed LVM than girls throughout childhood and adolescence.^15^ Our findings are consistent with the index proposed by Chirinos et al^6^ (ie, height^1.7^), based on a large sample of older men and women. Their study also highlighted a clear sex difference in LVM, even after accounting for allometric height. In keeping with the findings of their study, we found that pooling sexes resulted in a biased estimate of the allometric exponent for height (Figure 2A). Intriguingly, this biased estimate (2.68 in our study, 2.8–3.2 in Chirinos et al,^6^ depending on the sample studied) was close to the widely used index (height^2.7^) proposed by de Simone et al.^5^ The original data on which the height^2.7^ index is based were from 3 comparatively small samples with a wide range of ages and, although extensive sex analyses were performed, the existence of sex-interactions or a proportional bias between postpubertal young men and women was not explored.^5^ Not all previous studies in young people have supported this estimate. Foster et al^20^ analyzed the indexing of LVM relative to body size centile curves using height^2.7^ in healthy nonobese children and adolescents and observed a marked residual relationship with height, particularly in individuals <140 cm tall. This indicates that height^2.7^ did not fit these data well, yet unfortunately this study did not report sex-stratified analyses. Chinali et al^21^ studied a wide range of ages (0–18 years; n=400) and also concluded that height^2.7^ was inappropriate; they observed that LVM was most closely related to height^2.16^. Meanwhile, Daniels et al^22^ studied 192 children and adolescents (6–17 years; mean age 11.6 years) and reported that LVM/height^3^ was the most appropriate index for this age range. Analyses were stratified by both sex and ethnicity; however, based on a comparison with previously published data in adults^23^ that found exponents for height to be ≈2, they suggested that the allometric exponent for height might differ between children and adults.^22^ This could explain differences between their and other findings in children and our observations in older adolescents. While prepubescent boys and girls are known to have similar body composition, LV geometry and body composition are known to change considerably during aging over childhood,^14^ and it is plausible that this influences allometric exponents. In keeping with this, previous work has also shown that sex needs to be accounted for when scaling other physiological measures to body size.^24^

In its broadest sense, allometry refers simply to a deviation in the proportions of some organ from what would be geometrically predicted from isometric change.^25^ It has been suggested that allometric exponents may reflect the influences of biophysical factors (eg, the fractal-like structure of the vasculature and minimization of energy costs),^25^ although this proposal has been debated.^26^

We also investigated the allometric relationship between LVM and lean mass or BSA. Lean mass has been proposed as the optimal parameter for indexing LVM.^10^ In both sexes, an essentially linear relationship existed between lean mass and LVM, that is, the exponents in each sex were close to unity (1.15 [males]; 1.07 [females]). In addition, based on the (mostly) overlapping CIs between each sex for the exponents of the relationship between lean mass and LVM, there may be only a small sex difference when LVM is indexed to lean mass. Although we found no evidence that fat mass modified the associations between lean mass and LVM when all participants were pooled, when the sexes were considered separately, we saw evidence that fat modified the association between LVM and lean mass in males (Table S4). With increasing adiposity, the exponents for lean mass varied comparatively little by sex and, again, tended to stay close to 1 (Table S4), suggesting that the indexation of LVM to lean mass could be a preferable option. In agreement with our findings, it was found in participants of the MONICA study (aged 25–74 years) that sex differences disappeared when LVM was indexed to lean body mass,^11,27^ and also in other analyses of older adults with hypertension (aged 45–75 years).^28^

A disadvantage of lean mass is that it is not as easy to measure routinely as height or BSA. Consequently, researchers have often chosen to use height as an alternate indexing method.^29^ However, height may be an inadequate substitute for lean mass due to the variability in lean mass between subjects of the same height, with overweight individuals having both greater fat and lean mass compared with their counterparts of equal height.^30,31^ We observed that lean mass explained substantially more of the variance in LVM than height^1.7^, suggesting that height^1.7^ is an inferior index to lean mass at this age.

While the exponents for height and lean mass were similar between the sexes, those for BSA differed much more (1.53 [males]; 1.34 [females]). Exponents for BSA were not close to unity and the association between BSA and LVM was, therefore, nonlinear. Our results suggest that BSA perhaps should be raised to an allometric power if it is used to index LVM to body size; however it is unlikely that this would avoid the problems associated with use of BSA in overweight and obese individuals as these arise from the use of body weight in the formula.^5^ As with lean mass, when the sexes were considered separately, there was evidence that fat mass modified the associations with BSA in males. When we stratified by fat mass and by sex (Table S2), the sex-specific exponents changed considerably with increasing adiposity, supporting the view that, as in adults,^5^ BSA is an inappropriate method for indexation of LVM for older adolescents.

### Use of Different Indexation Methods for Detection of LVH

In older people, LVH predicts high risk for clinical events, irrespective of the presence of coronary artery disease or hypertension.^2^ While the application of all cutoffs showed that the majority of individuals in our sample did not have LVH, the κ statistics indicated only moderate agreement between cutoffs, and the detection rate varied considerably between each sex, with 8 to 77 females identified as possibly having LVH, compared with 6 to 44 males.

In adults the use of height^1.7^ as an index for LVM has been reported previously to be superior to height^2.7^ for the prediction of events,^6^ although the recent consensus document on hypertension in children and adolescents from the European Society of Cardiology acknowledges the uncertainty regarding best practice for normalizing LVM to body size in clinical practice in young people.^32^

Our study in adolescents lacks outcome data, so although we cannot determine which is the most appropriate method for detecting LVH in males and females at this age, based on our data, we suggest that sex-specific cutpoints may be required, particularly if height^1.7^ is used to index LVM.

### Limitations

This study has important limitations. The ALSPAC birth cohort is large but homogenous with respect to geographic origin, with a narrow range of ages (16.5–19.2 years; mean 17.69 [SD 0.32] years) studied in this particular analysis. Nevertheless, despite inevitable attrition over time, the sample remains reasonably representative of the UK population;^17^ with typical levels of obesity for United Kingdom for that time period.^33^ Most participants were adolescents of European heritage, and results may not be generalizable to other ethnicities or age groups. Measurements of LVM were made using 2D echocardiography, which makes geometric assumptions and is less accurate than other imaging methods such as magnetic resonance imaging.^31^ Nevertheless, LVM as assessed by echocardiography has good prognostic power^8^ and remains the most widely used method of cardiac imaging, being a good compromise between cost and quality.

Lastly, it should be noted that the estimate of the allometric exponent is dependent on model assumptions and may also depend on the fitting method used (ie, log-log linear versus 2 or 3 parameter nonlinear).^34^ Our approach, although widely used, may misestimate the exponent if our assumptions are invalid, despite fitting the data well.

### Perspectives

Common approaches for indexing LVM to body size, that is, LVM/height^2.7^ and LVM/BSA may be suboptimal in older adolescents. While lean mass was the best indexation measure in terms of explained variance in LVM, it may be difficult to measure in clinical practice, and indexation to height^1.7^ may be a practical alternative in adolescence. We also show current clinical reference values to identify LVH in adolescence are inconsistent and in particular, the detection rate when the current height^1.7^ cutoff is used varies substantially between each sex. This has the potential to result in high-risk individuals being misclassified, although the wider clinical significance of this remains to be established. This issue has been recognized as a complex and controversial one by the recent ESC consensus document on hypertension in children and adolescents.^32^ Further research should be undertaken in individuals from different ages and ethnicities, with longitudinal follow-up to ascertain which method of detection of LVH is the superior predictor of cardiovascular events.

## ARTICLE INFORMATION

### Acknowledgments

The authors are extremely grateful to all the families who took part in this study, the midwives for their help in recruiting them, and the whole ALSPAC (Avon Longitudinal Study of Parents and Children) team, which includes interviewers, computer and laboratory technicians, clerical workers, research scientists, volunteers, managers, receptionists and nurses.

### Sources of Funding

The UK Medical Research Council and Wellcome (grant ref: 217065/Z/19/Z) and the University of Bristol provide core support for ALSPAC (Avon Longitudinal Study of Parents and Children). This publication is the work of the authors and C.M. Park will serve as guarantor for the contents of this article. This research was funded in part, by the Wellcome Trust (grant number 092731). For the purpose of Open Access, the author has applied a CC BY public copyright license to any Author Accepted Manuscript version arising from this submission. A. Fraser and L.D. Howe are funded by UK Medical Research Council Post-doctoral research fellowships (MR/M009351/1/1 and MR/M020894/1, respectively). A.D. Hughes and N. Chaturvedi receive support from a Biomedical Research Center Award to University College London Hospital and a British Heart Foundation (BHF) Accelerator award to UCL (AA/18/6/34223). Additional funding for this study came from the Wellcome Trust (067100 and 086676/7/08/Z) and BHF (PG/06/145 and CS/15/6/31468 and SP/F/21/150020). N.J. Timpson is a Wellcome Trust Investigator (202802/Z/16/Z), the PI of the Avon Longitudinal Study of Parents and Children (MRC and WT 217065/Z/19/Z), is supported by the University of Bristol National Institute for Health and Care Research Biomedical Research Center, the MRC Integrative Epidemiology Unit (MC_UU_00011/1), and works within the Cancer Research UK Integrative Cancer Epidemiology Program (C18281/A29019).

### Disclosures

None.

### Supplemental Material

Figures S1–S3

Tables S1–S6

## Supplementary Material


