# Supplementary figures and images for: Is height2.7 appropriate for indexation of left ventricular mass in healthy adolescents? The importance of sex differences Left ventricular mass indexation
Source: Hypertension. Author manuscript; Available in PMC 2023 Oct 1. (PMC10510825; doi:10.1161/HYPERTENSIONAHA.121.17109)

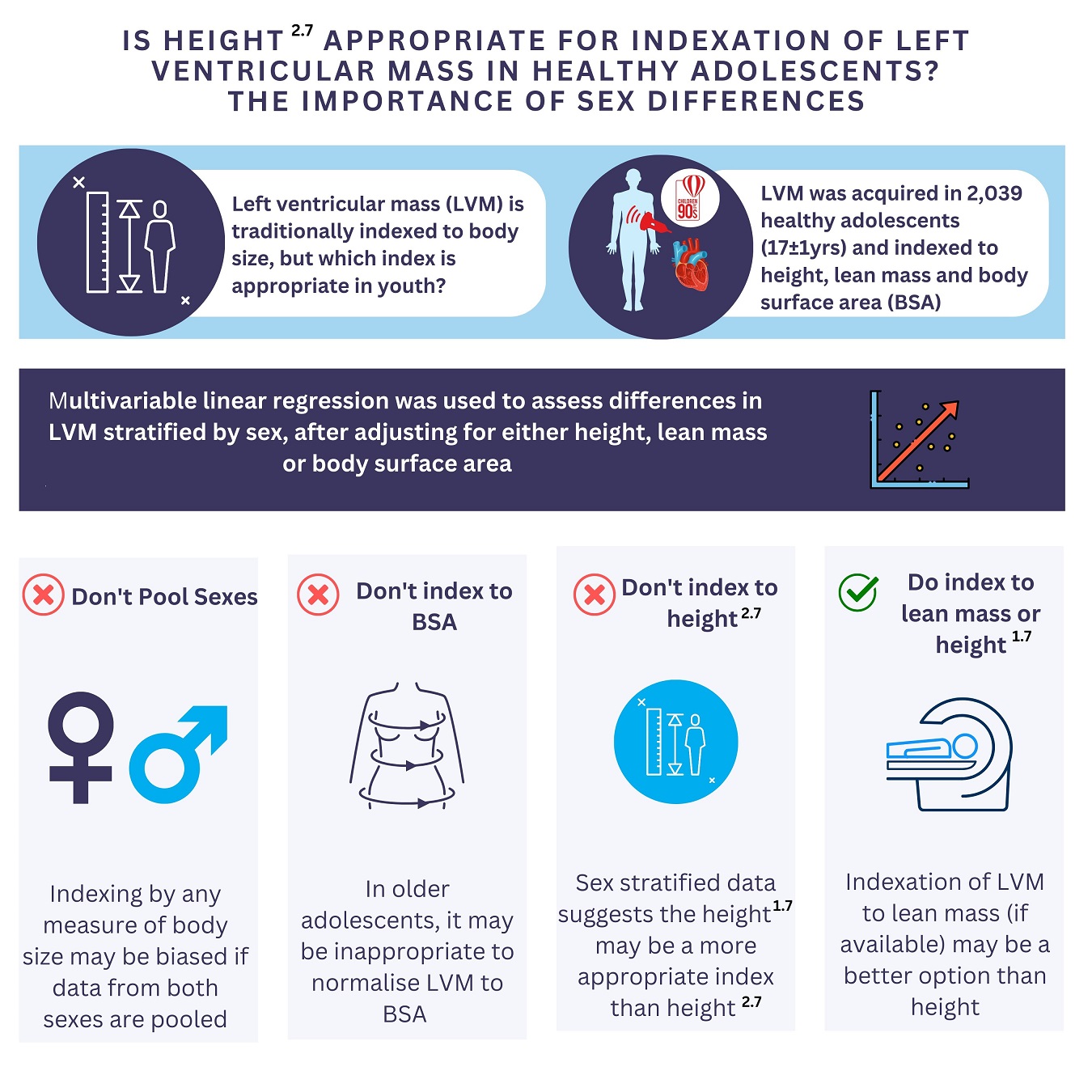

Supplement: Graphical Abstract [file EMS182008-supplement-Graphical_Abstract.jpg]
