# Supplementary material for: Is height2.7 appropriate for indexation of left ventricular mass in healthy adolescents? The importance of sex differences Left ventricular mass indexation
Source: Hypertension. Author manuscript; Available in PMC 2023 Oct 1. (PMC10510825; doi:10.1161/HYPERTENSIONAHA.121.17109)
Supplement: Supplemental Material (no PDF) [file EMS182008-supplement-Supplemental_Material__no_PDF_.doc]

# Is height^2.7^ appropriate for indexation of left ventricular mass in healthy adolescents? The importance of sex differences

# Supplemental material

Hannah C. M. TAYLOR^a,b^, MSc, PGCE, PhD, Nishi CHATURVEDI^a^, MBBS, MSc, MD, MRCP, George Davey SMITH^c,d^, MA, MD, BChir, MSc, Diana L. S. FERREIRA^c,d^, MSc, PhD, Abigail FRASER^c,d^, MA, MPH, PhD, Laura D. HOWE^c,d^, MSc, PhD, MSc, Alun D. HUGHES^a^, MBBS, PhD, FBPharmacolS, Debbie A. LAWLOR^c,d^, MBChB, MSc, PhD, Nic J. TIMPSON^c,d^, Chloe M. PARK^a^, BSc, PhD.

1. MRC Unit for Lifelong Health and Ageing, 5^th^ Floor, 1-18 Torrington Place, University College London, London, **UK**
2. **Oxford Population Health (NDPH), University of Oxford, Oxford, UK**
3. MRC Integrative Epidemiology Unit, University of Bristol, Bristol, UK
4. Bristol Population Health Science Institute, Bristol Medical School, University of Bristol, Bristol, UK

Author for correspondence:

Chloe Park, MRC Unit for Lifelong Health and Ageing, 5^th^ Floor, 1-18 Torrington Place, University College London, London W1T 7PH, UK

E-mail: chloe.park@ucl.ac.uk

Disclosures: None

Author for reprint requests:

Chloe Park, MRC Unit for Lifelong Health and Ageing, 5^th^ Floor, 1-18 Torrington Place, University College London, London W1T 7PH, UK

E-mail: [**chloe.park@ucl.ac.uk**](mailto:chloe.park@ucl.ac.uk)

Disclosures: None

## References

1. Lang RM, Badano LP, Mor-Avi V, Afilalo J, Armstrong A, Ernande L, Flachskampf FA, Foster E, Goldstein SA, Kuznetsova T. Recommendations for cardiac chamber quantification by echocardiography in adults: An update from the american society of echocardiography and the european association of cardiovascular imaging. *European Heart Journal-Cardiovascular Imaging*. 2015;16:233-271

2. de Simone G, Daniels SR, Devereux RB, Meyer RA, Roman MJ, de Divitiis O, Alderman MH. Left ventricular mass and body size in normotensive children and adults: Assessment of allometric relations and impact of overweight. *Journal of the American College of Cardiology*. 1992;20:1251-1260

3. Chirinos JA, Segers P, De Buyzere ML, Kronmal RA, Raja MW, De Bacquer D, Claessens T, Gillebert TC, St. John-Sutton M, Rietzschel ER. Left ventricular mass: Allometric scaling, normative values, effect of obesity, and prognostic performance. *Hypertension*. 2010;56:91-98

## Baseline characteristics for participants without measured LV mass

Table S1: Baseline characteristics of participants with measured left ventricular mass. HDL cholesterol = High-density Lipoprotein cholesterol; MVPA= Moderate to Vigorous Physical Activity. Asterisks indicate instances where t-test indicated a statistically significant difference (p<0.05) between those with and without LV mass.

|  | **All (n = 3066)** | | **Males (n = 1318)** | | **Females (n = 1748)** | |
| --- | --- | --- | --- | --- | --- | --- |
|  | **n** | **Mean ± St Dev**  **/Median +IQR** | **n** | **Mean ± St Dev**  **/Median +IQR** | **n** | **Mean ± St Dev**  **/Median +IQR** |
| ***Age (years)*** | 3066 | 17.9 ± 0.51 | 1318 | 17.9 ± 0.49 | 1748 | 17.9 ± 0.52 |
| ***Height (cm)*** | 2955 | 171.1 ± 9.29 | 1276 | 178.7 ± 6.77 | 1679 | 165.3 ± 6.27 |
| ***Weight (kg)*** | 2951 | 67.01 ± 14.3 | 1275 | 72.7 ± 13.9 | 1676 | 62.7 ± 13.15 |
| ***Body mass index (kg/m^2^)*** | 2950 | 22.8 ± 4.35 | 1275 | 22.7 ± 4.01 | 1675 | 22.9 ± 4.60 |
| ***Overweight (BMI 25-30 kg/ m^2^)*** | 445 |  | 194 |  | 251 |  |
| ***Obese (BMI >30 kg/ m^2^)*** | 311 |  | 120 |  | 191 |  |
| ***Total fat mass (kg)*** | 2763 | 16.2 (10.8, 22.96) | 1206 | 10.9 (6.91, 17.8) | 1557 | 19.1 (14.7, 25.3) |
| ***Total lean mass (kg)*** | 2763 | 45.6 ± 10.2 | 1206 | 55.4 ± 6.48 | 1557 | 37.96 ± 4.36 |
| ***Body surface area (BSA) (m^2^)*** | 2950 | 1.78 ± 0.199 | 1275 | 1.90 ± 0.18 | 1675 | 1.69 ± 0.16 |
| ***Systolic blood pressure (mmHg)*** | 2960 | 116.3 ± 11.4 | 1273 | 122.3 ± 10.7 | 1687 | 111.8 ± 9.79 |
| ***Diastolic blood pressure (mmHg)*** | 2960 | 64.7 ± 7.66 | 1273 | 64.47 ± 7.73 | 1687 | 64.8 ± 7.60 |
| ***Heart rate (bpm)*** | 2146 | 70.6 ± 11.00 | 938 | 67.4 ± 10.7* | 1208 | 73.03 ± 10.6* |
| ***Total cholesterol (mmol/l)*** | 1810 | 3.76 ± 0.70 | 856 | 3.54 ± 0.62 | 954 | 3.96 ± 0.71 |
| ***HDL cholesterol (mmol/l)*** | 1810 | 1.26 ± 0.30 | 856 | 1.17 ± 0.25 | 954 | 1.34 ± 0.31 |
| ***Triglycerides (mmol/l)*** | 1810 | 0.76 (0.6, 0.99) | 856 | 0.74 (0.59, 0.97) | 954 | 0.77 (0.61, 1.00) |
| ***MVPA at age 15 (minutes/day)*** | 1364 | 33.1 (18.7, 33.1) | 609 | 25.3 (14.7, 41.2) | 755 | 14.7 (7.00, 24.3) |
|  |  | | | | | |
| ***Smoking*** | **n** | **%** | **n** | **%** | **n** | **%** |
| *Never* | 1048 | 45.60 | 490 | 49.30 | 558 | 42.79 |
| *Ever* | 557 | 24.24 | 222 | 22.33 | 335 | 25.69 |
| *Current* | 693 | 30.16 | 282 | 28.37 | 411 | 31.52 |
| *Total* | 2298 | 100 | 994 | 100 | 1304 | 100 |
| ***Socioeconomic status*** |  |  |  |  |  |  |
| *I - Professional* | 285 | 10.5 | 132 | 11.25 | 153 | 9.92 |
| *II - Managerial and technical* | 1002 | 36.91 | 445 | 37.94 | 557 | 36.12 |
| *IIINM - Skilled non-manual* | 326 | 12.01 | 146 | 12.45 | 180 | 11.67 |
| *IIIM - Skilled manual* | 825 | 30.39 | 33 | 28.13 | 495 | 32.10 |
| *IV - Partly skilled* | 203 | 7.48 | 93 | 7.93 | 110 | 7.13 |
| *V - Unskilled* | 74 | 2.73 | 27 | 2.30 | 47 | 3.05 |
| *Total* | 2715 | 100 | 1173 | 100 | 1542 | 100 |

## Allometric relationships between LV mass and height, lean mass and body surface area

|  | | **Height** | |  | **Lean mass** | |  | **BSA** | | |
| --- | --- | --- | --- | --- | --- | --- | --- | --- | --- | --- |
|  |  | **n** | **Coefficient (+ 95% CI)** | **r^2^** | **n** | **Coefficient (+ 95% CI)** | **r^2^** | **n** | **Coefficient (+ 95% CI)** | **r^2^** |
| **Unstratified** | **Group** | 2039 | 2.68  (2.51, 2.84) | 0.32 | 2039 | 0.87  (0.84, 0.91) | 0.52 | 2039 | 1.74  (1.66, 1.81) | 0.51 |
|  | **Male** | 908 | 1.66  (1.30, 2.03) | 0.08 | 908 | 1.15  (1.04, 1.26) | 0.32 | 908 | 1.53  (1.39, 1.66) | 0.36 |
|  | **Female** | 1131 | 1.59  (1.27, 1.90) | 0.08 | 1131 | 1.07  (0.97, 1.16) | 0.31 | 1131 | 1.34  (1.23, 1.45) | 0.35 |
| **Total fat mass**  **1^st^ Quartile** | **Group** | 510 | 2.75  (2.33, 3.16) | 0.25 | 510 | 1.05  (0.96, 1.15) | 0.51 | 509 | 1.96  (1.77, 2.14) | 0.47 |
|  | **Male** | 227 | 1.77  (0.94, 2.60) | 0.07 | 227 | 1.31  (1.09, 1.53) | 0.38 | 227 | 1.99  (1.59, 2.39) | 0.30 |
|  | **Female** | 283 | 0.90  (0.27. 1.52) | 0.03 | 283 | 0.94  (0.74, 1.15) | 0.23 | 283 | 1.20  (0.84, 1.56) | 0.13 |
| **Total fat mass**  **2^nd^ Quartile** | **Group** | 510 | 3.08  (2.78, 3.38) | 0.44 | 510 | 0.99  (0.92, 1.05) | 0.66 | 510 | 1.90  (1.76, 2.04) | 0.60 |
|  | **Male** | 227 | 1.64  (0.94, 2.34) | 0.09 | 227 | 1.29  (1.09, 1.50) | 0.41 | 227 | 1.96  (1.58, 2.33) | 0.31 |
|  | **Female** | 283 | 1.31  (0.75, 1.87) | 0.07 | 283 | 0.92  (0.75, 1.09) | 0.29 | 283 | 1.59  (1.24, 1.93) | 0.22 |
| **Total fat mass**  **3^rd^ Quartile** | **Group** | 510 | 2.66  (2.31, 3.01) | 0.31 | 510 | 0.94  (0.87, 1.01) | 0.56 | 510 | 1.92  (1.75, 2.09) | 0.49 |
|  | **Male** | 227 | 1.15  (0.53, 1.77) | 0.06 | 227 | 0.87  (0.69, 1.05) | 0.28 | 227 | 1.48  (1.11, 1.84) | 0.22 |
|  | **Female** | 283 | 1.11  (0.56, 1.66) | 0.05 | 283 | 0.84  (0.66, 1.03) | 0.22 | 283 | 1.40  (1.00, 1.79) | 0.15 |
| **Total fat mass**  **4^th^ Quartile** | **Group** | 509 | 2.78  (2.46, 3.11) | 0.36 | 509 | 0.89  (0.82, 0.97) | 0.52 | 509 | 1.71  (1.55, 1.86) | 0.48 |
|  | **Male** | 227 | 1.43  (0.78, 2.07) | 0.08 | 227 | 0.83  (0.62, 1.04) | 0.21 | 227 | 1.13  (0.83, 1.43) | 0.19 |
|  | **Female** | 283 | 1.53  (0.97, 2.10) | 0.09 | 283 | 0.81  (0.63, 0.98) | 0.23 | 283 | 1.25  (0.98, 1.52) | 0.22 |

Table S2: Allometric relationships between left ventricular mass (LV mass) and height, lean mass and body surface area (BSA), stratified by sex and fat mass quartiles and presented with 95% confidence intervals and r^2^ values. All p values <0.001 with the exception of the female result for the 1st fat mass quartile for height (p=0.005).

## Use of different indexing methods and cut offs to identify individuals with LVH

|  | **Male** | | | | **Female** | | |
| --- | --- | --- | --- | --- | --- | --- | --- |
| *Absolute LV mass, g*  *(linear ASE cut-off from ^1^)* |  | **n** | **%** |  | | **n** | **%** |
|  | **<224** | 894 | 98.5 | **<162** | | 1104 | 97.6 |
|  | **>224** | 14 | 1.5 | **>162** | | 27 | 2.4 |
|  | **Total** | 908 | 100 | **Total** | | 1131 | 100 |
|  |  |  |  |  | |  |  |
| *Absolute LV mass, g*  *(2D ASE cut-off from ^1^)* |  | **n** | **%** |  | | **n** | **%** |
|  | **<200** | 864 | 95.2 | **<150** | | 1077 | 95.2 |
|  | **>200** | 44 | 4.8 | **>150** | | 54 | 4.8 |
|  | **Total** | 908 | 100 | **Total** | | 1131 | 100 |
|  |  |  |  |  | |  |  |
| *LV mass/BSA, g/m^2^*  *(linear ASE cut-off from ^1^)* |  | **n** | **%** |  | | **n** | **%** |
|  | **<115** | 898 | 98.9 | **<95** | | 1118 | 98.9 |
|  | **>115** | 10 | 1.1 | **>95** | | 13 | 1.1 |
|  | **Total** | 908 | 100 | **Total** | | 1131 | 100 |
|  |  |  |  |  | |  |  |
| *LV mass/BSA, g/m^2^*  *(2D ASE cut-off from ^1^)* |  | **n** | **%** |  | | **n** | **%** |
|  | **<102** | 874 | 96.3 | **<88** | | 1101 | 97.3 |
|  | **>102** | 34 | 3.7 | **>88** | | 30 | 2.7 |
|  | **Total** | 908 | 100 | **Total** | | 1131 | 100 |
|  |  |  |  |  | |  |  |
| *LV mass/height^2.7^, g/m^2.7^*  *(cut-off from ^2^)* |  | **n** | **%** |  | | **n** | **%** |
|  | **<50** | 902 | 99.3 | **<47** | | 1123 | 99.3 |
|  | **>50** | 6 | 0.7 | **>47** | | 8 | 0.7 |
|  | **Total** | 908 | 100 | **Total** | | 1131 | 100 |
|  |  |  |  |  | |  |  |
| *LV mass/height^1.7^, g/m^1.7^*  *(cut-off from ^3^)* |  | **n** | **%** |  | | **n** | **%** |
|  | **<81** | 895 | 98.6 | **<60** | | 1054 | 93.2 |
|  | **>81** | 13 | 1.4 | **>60** | | 77 | 6.8 |
|  | **Total** | 908 | 100 | **Total** | | 1131 | 100 |
|  |  |  |  |  | |  |  |

Table S3: Application of different clinical cut-offs in detecting the presence of left ventricular hypertrophy (LVH).

## Effect modifications

|  | **P value** | | |
| --- | --- | --- | --- |
| **By sex** | **All (n= 2039)** | **Males (n=908)** | **Females (n=1131)** |
| **LVM and height** | 0.044 |  | |
| **LVM and lean mass** | <0.001 |
| **LVM and BSA** | <0.001 |
|  |  | | |
| **By fat mass (continuous)** |
| **LVM and height** | 0.567 | 0.407 | 0.100 |
| **LVM and lean mass** | 0.407 | <0.001 | 0.065 |
| **LVM and BSA** | 0.100 | <0.001 | 0.999 |
|  |  | | |
| **By fat mass (quartile)** |
| **LVM and height (q1)** |
| **LVM and height (q2)** | 0.072 | 0.861 | 0.294 |
| **LVM and height (q2)** | 0.484 | 0.755 | 0.430 |
| **LVM and height (q2)** | 0.023 | 0.746 | 0.011 |
|  |  | | |
| **By fat mass (quartile)** |
| **LVM and lean mass (q1)** |
| **LVM and lean mass (q2)** | 0.824 | 0.722 | 0.854 |
| **LVM and lean mass (q2)** | 0.956 | 0.146 | 0.984 |
| **LVM and lean mass (q2)** | 0.939 | 0.085 | 0.695 |
|  |  | | |
| **By fat mass (quartile)** |
| **LVM and BSA (q1)** |
| **LVM and BSA (q2)** | 0.459 | 0.792 | 0.184 |
| **LVM and BSA (q2)** | 0.543 | 0.367 | 0.426 |
| **LVM and BSA (q2)** | 0.896 | 0.008 | 0.509 |
|  |  | | |

Table S4: Effect modifications of the associations between left ventricular mass and height, lean mass and BSA by sex and fat mass. q = quartile; BSA = body surface area.

## Allometric relationships between LV mass and height, lean mass and body surface area –additional sensitivity analyses

|  | **Height** | | | **Lean mass** | | | **BSA** | | |
| --- | --- | --- | --- | --- | --- | --- | --- | --- | --- |
|  | **n** | **Coefficient**  **(+ 95% CI)** | **r^2^** | **n** | **Coefficient**  **(+ 95% CI)** | **r^2^** | **n** | **Coefficient**  **(+ 95% CI)** | **r^2^** |
| **All** | 1913 | 2.72  (2.55, 2.89) | 0.34 | 1913 | 0.87  (0.83, 0.90) | 0.52 | 1913 | 1.85  (1.75, 1.91) | 0.51 |
| **Male** | 869 | 1.68  (1.31, 2.05) | 0.08 | 869 | 1.14  (1.03, 1.25) | 0.32 | 869 | 1.61  (1.46, 1.76) | 0.34 |
| **Female** | 1044 | 1.52  (1.21, 1.84) | 0.08 | 1044 | 1.00  (0.90, 1.10) | 0.27 | 1044 | 1.36  (1.23, 1.48) | 0.30 |

Table S5: Allometric relationships between left ventricular mass (LV mass) and height, lean mass and body surface area (BSA and presented with 95% confidence intervals and r^2^ values. Participants with obesity (n=126; obesity was defined as body mass index >30 kg/m^2^) were removed from analysis. All p values <0.001.

|  | **Height** | | | **Lean mass** | | | **BSA** | | |
| --- | --- | --- | --- | --- | --- | --- | --- | --- | --- |
|  | **n** | **Coefficient**  **(+ 95% CI)** | **r^2^** | **n** | **Coefficient**  **(+ 95% CI)** | **r^2^** | **n** | **Coefficient**  **(+ 95% CI)** | **r^2^** |
| **All** | 1566 | 2.70  (2.52, 2.88) | 0.35 | 1566 | 0.85  (0.81, 0.89) | 0.53 | 1566 | 1.85  (1.75, 1.94) | 0.49 |
| **Male** | 726 | 1.73  (1.34, 2.12) | 0.09 | 726 | 1.12  (1.01, 1.24) | 0.32 | 726 | 1.64  (1.45, 1.82) | 0.30 |
| **Female** | 840 | 1.40  (1.07, 1.74) | 0.07 | 840 | 0.96  (0.84, 1.07) | 0.25 | 840 | 1.26  (1.10, 1.42) | 0.22 |

Table S6: Allometric relationships between left ventricular mass and height, lean mass and body surface area (BSA) and presented with 95% confidence intervals and r^2^ values. Participants with obesity, or who were classified as overweight, (n=473; overweight was defined as body mass index >25 kg/m^2^) were removed from analysis. All p values <0.001.

## Scatterplot for fat mass


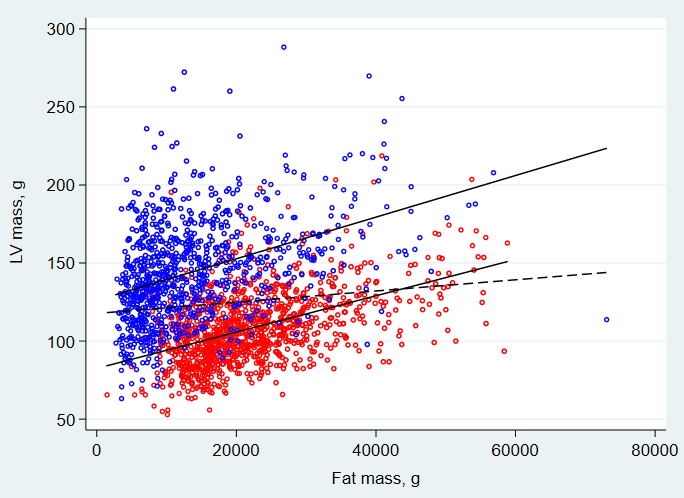


Figure S1: Scatterplot showing the relationship between fat mass and LV mass (untransformed) for males (blue circles) and females (red circles). The solid black lines indicate the gradient for each sex separately, while the dashed line indicates the gradient for the pooled group.

## Additional scatterplots for log–transformed lean mass (sex-stratified)


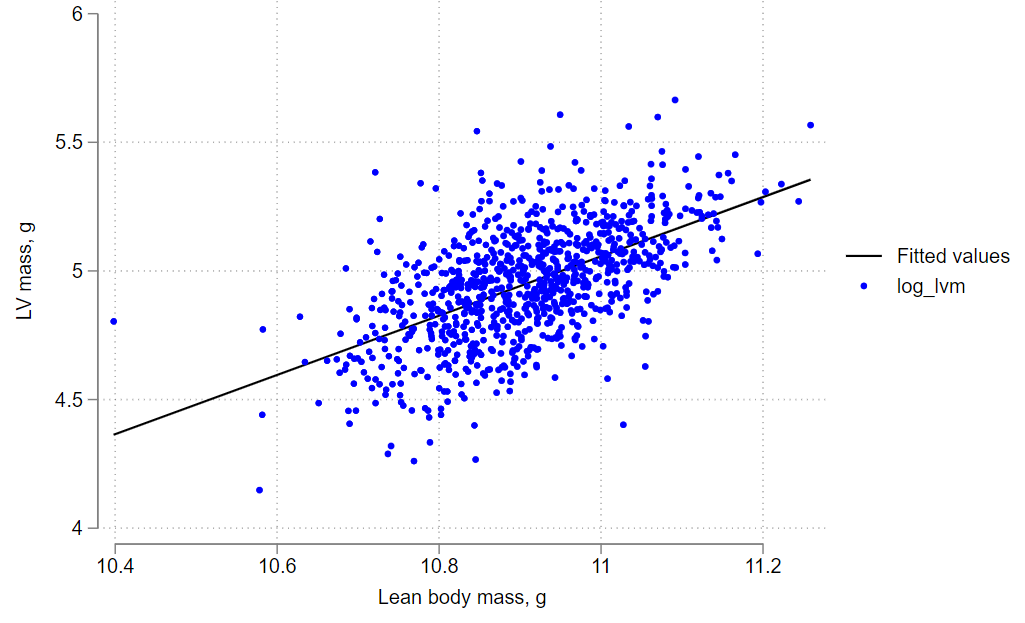


Figure S2: Scatterplot showing the relationship between log-transformed lean body mass and LV mass (untransformed) in males only. The solid black line indicates the gradient.


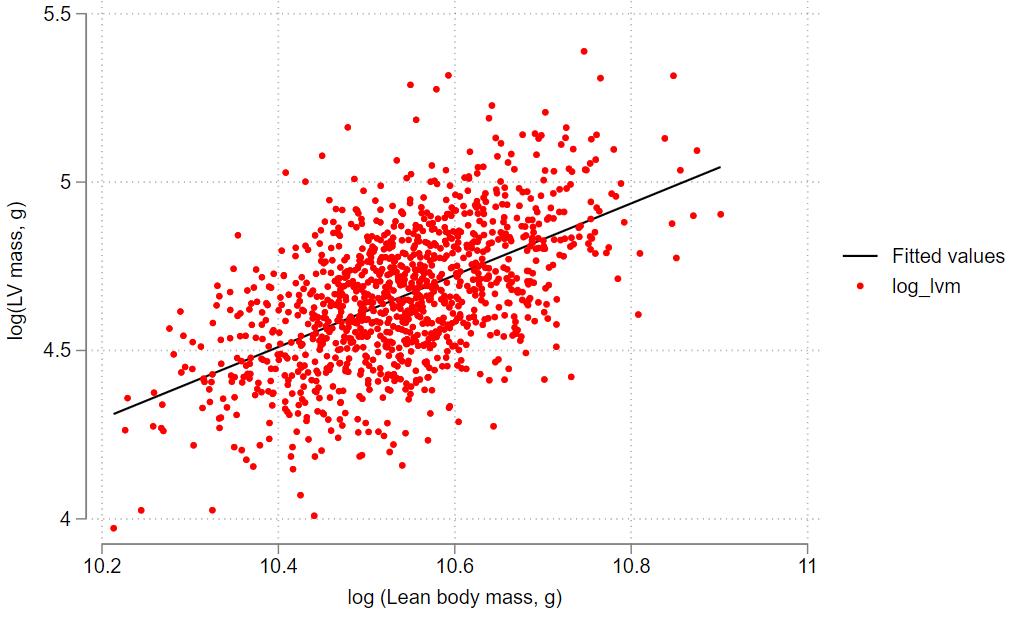


Figure S3: Scatterplot showing the relationship between log-transformed lean body mass and LV mass (untransformed) in females only. The solid black line indicates the gradient.
